# Enhancing Stability of Destabilized Green Fluorescent Protein Using Chimeric mRNA Containing Human Beta-Globin 5′ and 3′ Untranslated Regions

**Published:** 2019

**Authors:** Setare Adibzadeh, Majid Fardaei, Mohammad Ali Takhshid, Mohammad Reza Miri, Gholamreza Rafiei Dehbidi, Ali Farhadi, Reza Ranjbaran, Parnian Alavi, Negin Nikouyan, Noorossadat Seyyedi, Samaneh Naderi, Alireaz Eskandari, Abbas Behzad-Behbahani

**Affiliations:** 1. Diagnostic Laboratory Sciences and Technology Research Center, Faculty of Paramedical Sciences, Shiraz University of Medical Sciences, Shiraz, Iran; 2. Department of Medical Biotechnology, Faculty of Paramedical Sciences, Shiraz University of Medical Sciences, Shiraz, Iran; 3. Department of Genetics, Faculty of Medicine, Shiraz University of Medical Sciences, Shiraz, Iran

**Keywords:** Beta-globins, Genetic therapy, Green fluorescent proteins, Half-life, mRNA

## Abstract

**Background:**

In spite of recent progress in mRNA technologies and their potential applications for treatment of human diseases, problems such as the transient nature of mRNA limit the stability of gene up-regulation and, thus, potentially reduce mRNA efficiency for gene therapy. Using human β-globin 5′ and 3′ untranslated regions (UTRs), this study aimed to develop the different chimeric constructs of mRNAs to increase the stability of destabilized green fluorescent protein (EGFPd2) in HEK 293 cells.

**Methods:**

Purified human β-globin (HBG) 5′-3′UTRs, and the coding sequence of destabilized green fluorescent protein (EGFPd2) were amplified separately and ligated to each other using SOEing PCR method in a different format. As controls, the original construct of EGFPd2 under the control of T7 promoter was used. Following *in vitro* transcription, HEK 293 cells were then transfected with several constructs and incubated at 37°*C* in a CO_2_ incubator. They were monitored under a fluorescence microscope every four hours for the first 24 *hr*, then every 12 *hr* afterwards. The resulting fluorescence was measured as a surrogate for translation efficiency and duration.

**Results:**

By monitoring the HEK cells over 48 *hr*, cells transfected with mRNA with various HBG UTRs showed significantly different fluorescence intensity and stability in comparison with the pEGFPd2 prototype (control transcript) overtime. Overall, the images show that replacement of the 3′ UTR end of the prototype vector pGFPd2 with the 3′ end of β-globin mRNA increases the half-life of the chimeric mRNA for more than 32 *hr*.

**Conclusion:**

This result indicates that β-globin 3′ UTR would definitely increase the half-life of mRNA and may help to decrease the mRNA therapeutic dosage in the treatment of diseases associated with mRNA therapy.

## Introduction

Gene therapy, as a new strategy for the treatment of non-infectious diseases such as neurological disorders and cancer or even inherited disorders, is a promising technology 1. However, for the transfer and introduction of a functional gene into the target cells, plasmid DNA or virus vectors containing the gene of interest are usually employed. In this case, accidental crossing over between the donor of DNA and recipient DNA can be expected. Furthermore, induction of mutations in another part of the recipient genome may occur 2.

Messenger RNA (mRNA) on the other hand, as a new system of gene therapy, could offer a relatively safe and efficient alternative technique 3,4. However, the half-lives of the wild-type of mRNA molecules are relatively short and they are rapidly degraded in the body; they also strongly stimulate the immune system. Therefore, in order to increase the stability and to promote efficient mRNA therapy, some modification in the construction of mRNA needs to be applied properly before *in vivo* administration.

Overall, the stability or instability of the mRNA depends on the rate of synthesis of the cell nucleus and its cytoplasmic degradation 5,6. In prokaryotes, the half-life of mRNA is about minutes 7,8 while in eukaryotes, it is generally a few hours 9. In eukaryotic cells, the rate of mRNA degradation depends on deadenylation at the 3′ end, decapping at the 5′ end, and intracellular endonucleolytic activity of nucleases to initiate cleavage and to remove part of the mRNA along with the 3′ poly (A) tail 10,11. Adenylate-uridylate (AU)-rich instability elements, which are typically found in the 3′ untranslated region (3′UTR), the region between the stop codon and the start of the poly(A) tail of the most mammalian cells, are recognized by RNA binding proteins that impact mRNA deadenylation and subsequently leading to mRNA degradation 12,13.

Specifically, the 3′ UTR is known to play a major role in mRNA stabilization where the stability determinants are not subjected to disruption by an actively translating ribosome 14.

Human globin mRNAs are highly stable mRNAs and have been known to have half-lives that range from 10 *hr* to 48 *hr*, which is a useful attribute for mRNA therapy 15,16.

Furthermore, for the development and the stability of mature mRNA, nuclear cleavage and addition of poly (A) tail at the 3′ end of the pre-mRNA is a crucial step. For the protection of the 3′ end against degradation by exonucleases and the export of mature mRNA to the cytoplasmic environment, nuclear polyadenylation is a crucial step 17. However, cytoplasmic polyadenylation was shown to play an important role in gene expression as well 18.

The 5′ untranslated region (5′ UTR) of messenger RNA has been shown to perform an important regulatory function in post-transcriptional processes 19. A 7-methyl-guanosine at their 5′ ends (5′ cap) is another important structure for the expression and the stability of mRNA in cell cytoplasm when mRNA leaves the nucleus.

Overall, increasing the stability of mRNA for the production of a specific protein is a very important feature for mRNA therapy. Accordingly, this study was designed to investigate whether different combinations of UTRs from long lived cellular transcript would be able to confer increased stability and translation efficiency when combined with other gene sequences such as destabilized green fluorescent protein (*EGFPd2*) gene which has a fluorescence half-life between 2 to 10 *hr* 20. The functionality of the recombinant EGFPd2 mRNA constructs was verified by following the fluorescence intensity over time.

## Materials and Methods

### Strains and plasmids used in the study

Vector pBHA containing synthetic nucleotide sequences of 5′ and 3′ beta globin UTRs, T7 promoter which allows for *in vitro* transcription of cloned gene, and Kozak nucleotide sequence were obtained from Bioneer Corporation (South Korea). The pBHA also contained the ampicillin-resistance gene for selection in *Escherichia coli* (*E. coli)*. Plasmid contained the destabilized GFP (pCAG-EGFPd2) was a gift from Connie Cepko (Addgene plasmid # 14760). The *E. coli* strain DH5α was used for plasmid transformation and amplification.

### Designing PCR primers

AlleleID 7.5 software was used to design primers for the PCR amplification and detection of different DNA fragments. The sequences of the primers, which were originally designed for the gene splicing by overlap extension (SOEing) PCR technique, are given in table 1, where the reverse and forward primers of 5′UTR and 3′UTR had complement sequences with 5′ and 3′ of the coding sequence of EGFPd2. The specificity of the primers was confirmed using Primer-BLAST (www.ncbi.nlm.nih.gov/tools/primer-blast) for rapid comparison of nucleotide sequences.

**Table 1.** Primers designed for the amplification of UTRs and GFPds sequences

**Primer names**	**Forward sequence 5′-3′ (F1)**	**Reverse sequence 5′-3′ (R1)**
**EGFPd2**	AACAGACACCATGGTGAGCAAGGGCGAGG	GAAAGCGAGC**CTACACATTGATCCTAGCAGAAGC**
**3′UTR**	**ATCAAGATAA**GCTCGCTTTCTTGCTGTCC	GCAATGAAAATAAATGTTTT
**5′UTR**	GATCATTAATACGACTC	**TGCTCACCAT**GGTGTCTGTTTGAGGTTGC

Designed primers for SOEing PCR: Bold and underline sequences are complementary to EGFPd2 sequence of the plasmid, pale sequence complementary to β-globin UTRs.

### Creating DNA templates and purification of fragments

Using plasmids pBHA and pCAG-EGFPd2 (a gift from Connie Cepko) 21 and Addgene plasmid # 14760 as the templates, human β-globin (HBG) 5′-3′UTRs, and the coding sequence of destabilized green fluorescent protein (EGFPd2) were amplified by specific primers. PCR and SOEing reactions were carried out in separate tubes. Initially, HBG5′-3′ UTRs, and the coding sequence of EGFPd2 were amplified by simple PCR in a thermal cycler for 40 cycles, each consisting of 1 *min* at 94*°C*, 1 *min* at 43*°C* for 3′UTR, 47*°C* for 5′UTR, and 67*°C* for EGFPd2 coding sequences, respectively, 1 *min* at 72*°C* and a final extension for 10 *min*. An assay reaction was performed in a final volume of 25 *μl* in 50 *mM* KCl, 10 *mM* Tris-Cl, pH=8.3, 1.5 *mM* MgCl_2_, 200 *μM* dNTPs, 0.4 *μM* of each primer, and 2.5 units Taq polymerase. Amplified PCR products were determined on 1.5% agarose gel in TAE buffer and the gels were stained with ethidium bromide and photographed.

### Construction of recombinant replicons using SOEing PCR

DNA from the appropriate bands generated by simple PCR was recovered from the gel fragment by *AccuPrep*^®^ Gel Purification Kit (Bioneer, South Korea). Using the primers given in table 1, a 1078*bp* DNA fragment (5′ HBG-UTR-EGFPd2-3′HBG UTR) was constructed by SOEing PCR method as previously described 22. In addition, Cap-5′ UTR –EGFPd2-3′ HBG UTR–Poly A Tail was also constructed. As the controls, the original construct of EGFPd2 without the beta globin UTRs under the control of T7 promoter were used.

### T/A cloning and in vitro transcription

The final recombinant products were gel-purified (Bioneer, South Korea) and were inserted into the PTZ57R/T vector using a T/A cloning kit (InsTA-clone^TM^ PCRcloning kit, MBI Fermentas) according to the manufacturers' protocols. The resulting recombinant DNA plasmids were transformed into competent cells of *E. coli* and plated onto an LB agar plate supplemented with ampicillin, IPTG, and X-gal and were grown overnight at 37°*C*. Colony PCR was performed on white colonies to quickly screen for plasmids containing the desired inserts. To confirm the correct sequence of the inserts, DNA sequencing was conducted on purified plasmids as well.

*In vitro* transcription was performed by HiScribe^™^ T7 High Yield RNA Synthesis Kit (New England Biolabs) according to the manufacturer's protocols. All transcripts were capped with 3′-0-Me-m^7^G(5′)ppp(5′)G RNA cap structure analogue. Poly (A) Tailing kits (New England Biolabs) were used to add about 150–200 nucleotide (A) tail to transcripts. mRNAs were then purified with the RNeasy Mini Kit (Qiagen, Germany). The concentration of mRNA was quantified by the absorbance at 260 *nm* with a NanoDrop spectrophotometer.

### mRNA transfection into HEK 293 line

HEK 293 cells cultured in DMEM supplemented with L-Glutamine and 10% fetal bovine serum were grown at 37°*C* with 5% CO_2_. After 80–90% confluence on the day of transfection, 4×10^5^ cells/well were seeded in 24-well plates. For each well, 0.75 *μg* of mRNA was mixed with Opti-MEMOpti-MEM (Invitrogen) medium in a total of 250 *μl*. The mixture was then combined with a solution of 50 *μl* Lipofectamine^™^ 2000 (Invitrogen) and incubated at room temperature for 30 *min* to generate the RNA-Lipofectamine complex. After a 30 *min* incubation, 50 *μl* of the mRNA-Lipofectamine complexes were directly added to each well containing HEK 293 cells and incubated at 37*°C* in a CO_2_ incubator and monitored under a fluorescence microscope (Zeiss, Germany) every four *hr* for the first 24 *hr* and then cells were measured every 12 *hr* afterwards. The expression duration and fluorescence intensity of EGF-Pd2 were measured by fluorescence microscopy.

### Normalization of the procedure

The scale of GFP expression was normalized using total cell numbers transfected with the same concentration of various constructs at the same conditions. Ten visual fields from different areas of each flask transfected with different constructs were evaluated by two people independently (blind to experiment). Positive expressions were calculated as percentage of positive cells per field (%) and normalized by the total cell number in each field.

## Results

### Construction of chimeric EGFPd2 gene

Human β-globin 5′ and 3′, T7 promoter, and Coding Sequence (CDS) of EGFPd2 sequences were initially amplified in separate assays and the amplicons were then ligated together in the correct orientation by gene SOEing PCR using the primers listed in table 1. The chimaera was subsequently cloned into the PTZ57R/T vector for *in vitro* transcription.

### Creating the mRNA templates by in vitro transcription

**Figure 1.**
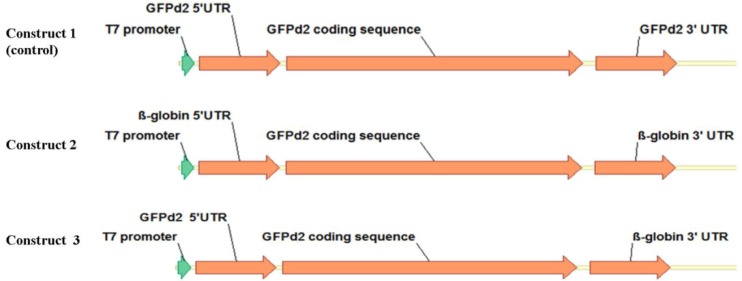
Schematic presentation of template constructs, consisting of a T7 promoter, a 5′ UTR, a EGFPd2 coding region, and a 3′ UTR.

To generate different mRNA transcripts, three different constructs were produced for *in vitro* transcription. A schematic illustration of the building blocks of all constructs is shown in figure 1.

### Fluorescent microscopy assessment

**Figure 2.**
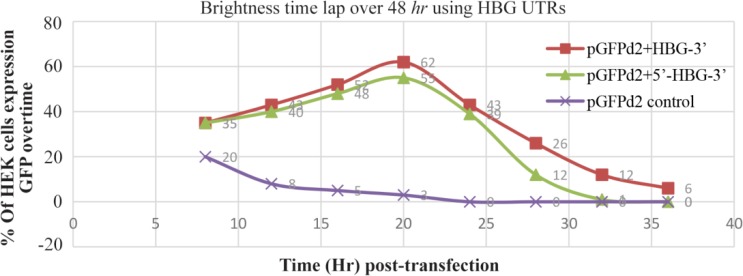
The comparison of cell transfection and fluorescence intensity of each mRNA over time. Constructs with HBG-3′ UTRs have the highest fluorescence level 20 *hr* post-transfection.

By monitoring the HEK cells over 48 *hr*, cells transfected with mRNA with various HBG UTRs showed significantly different fluorescence intensity and stability in comparison with the pEGFPd2 prototype (control plasmid) over time. All those with RNA replaced with beta globin UTRs reached a peak in fluorescent level at about 15 *hr* before subsequent decreasing, once the mRNA had all been translated and expressed in protein (Figure 2).

**Figure 3.**
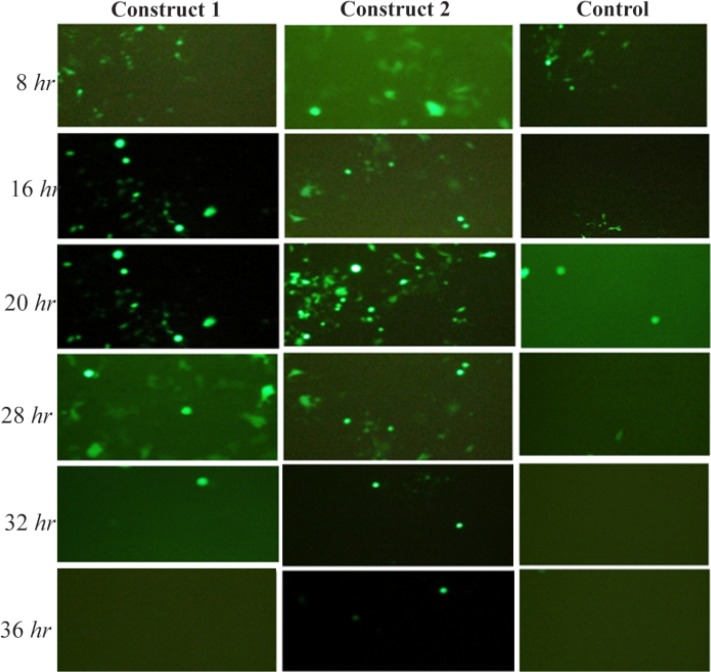
Fluorescence intensity and percentage of HEK 293 cells expressing GFP with HBG UTRs overtime. Construct 1: Cap-5′ HBG UTR –EGFPd2-3′ HBG UTR – poly A tail Construct 2: Cap-5′ UTR –EGFPd2-3′ HBG UTR – poly A tail Control: Cap-5′UTR –EGFPd2-3′ UTR-poly A tail.

HEK293 cells transfected with constructs containing both 5′-3′ β globin UTRs showed green fluorescent light stability for 28 *hr* with a maximum fluorescence intensity 20 *hr* post-transfection. Same results were obtained when the cells were transfected with a construct containing human 3′ β globin UTR but 5′ UTR of the original plasmid vector (Construct 2). However, the fluorescence intensity of construct 2 was stable for 36 *hr* post transfection (Figure 3). On the other hand, the highest fluorescence intensity level was observed 8 *hr* post-transfections in only 20% of the cells transfected with the prototype plasmid vector pGFPds and declined after 20 *hr* of incubation. Overall, the percentage of cells transfected with plasmid vectors containing HBG-3′ was higher in comparison with other constructs.

## Discussion

It is proposed that RNA therapy is able to provide efficient expression of the protein of interest in a cell and would be an alternative method for heterologous DNA gene therapy which has, however, several problems including chromosomal integration resulting in alteration or damage to the host cell genomic DNA 23. On the other hand, some alterations in mRNA 3′ or 5′ UTRs might be an effective way to increase the stability of mRNA against intracellular nucleases and to promote translation efficiency of the protein of interest.

The Untranslated Regions (UTRs), in particular, the 3′ ends of mRNA transcripts, contain important sequences that influence the post-transcription efficiency of mRNA in the cell 24.

The most well-recognized examples of such UTR mRNA are the alpha- and β-globin mRNAs with the half-lives of 16 to 48 *hr* in adult and neonatal reticulocytes, which are useful for mRNA therapy 25.

The present study aimed at improving the efficiency of mRNA by replacing 5′-3′ UTR sequences of EGF-Pd2 with the UTRs of human beta globin. Moreover, to increase the nuclease stability of the mRNA, a 5′ diguanosine (7 m) cap and a poly A tail were successfully added to the constructs.

Whereas the wild-type GFP has a half-life of about 26 *hr*, the protein half-life of EGFPd2, which contains the EGFP coding sequence fused to the C-terminal sequence of ornithine decarboxylase (amino acids 422–461) is between 2 to 10 *hr* 20. To increase the efficiency and the stability of EGFPd2, using SOEing PCR technique, EGFPd2 UTRs were replaced with β-globin UTRs. 7-methyl-guanosine (7mG) cap and a poly (A) tail, which physically protect the mRNA ends from exonucleolytic decay and also serve to recruit translation initiation machinery were also added to 5′ and 3′ ends of the constructs. Overall, three types of mRNAs were constructed by *in vitro* transcription. Based on fluorescence measurement, after mRNA transfection into the HEK 293 cells, construct Cap-5′ UTR–EGFPd2-3′ HBG UTR–poly A tail showed the highest expression level of fluorescent and high stability of all the mRNAs, followed by Cap-5′ HBG UTR–EGFPd2-3′ HBG UTR–poly A tail. However, construct cap-5′ UTR–EGFPd2-3′ UTR-poly A tail as the control showed the low expression level and stability of GFP over time.

Monitoring green fluorescent protein synthesis in single cells indicated that the time elapsed until the diminishing of the fluorescent light in a cell transfected with chimeric mRNAs compared to the control group was about 16 *hr*. In other words, the stability of GFP in the cells transfected by chimeric mRNAs was four times higher than the control group. However, the time interval between the recovery of fluorescence of the two chimeric mRNA constructs in single cells was about four *hr*. The stability of GFP in transfected cells with either construct 1 or 2 containing 3′ HBG UTRs was almost the same, which indicates that 3′ HBG UTR region would be able to strengthen the stability of mRNAs.

It has been shown that the β-globin 3′ UTR harbours a stability element that maps to a 14-nt pyrimidine-rich element or PRE with specific recognition sites for RNA-binding proteins that are involved in regulating mRNA stability. Site-specific PRE mutations can destabilize globin mRNA 26,27. Therefore, in mRNA therapy, replacement of the original sequence of 3′ UTR of a gene with the wild type of β-globin 3′ UTR may increase the stability of mRNA and consequently improve the expression of a desirable protein.

## Conclusion

In conclusion, these results indicate that β-globin 3′ UTR would definitely increase the half-life of destabilized GFP mRNA and therefore may be helpful in decreasing further therapeutic dosage of mRNA in the treatment of diseases associated with mRNA therapy in future.
